# A Forest of Sub-1.5-nm-wide Single-Walled Carbon Nanotubes over an Engineered Alumina Support

**DOI:** 10.1038/srep46725

**Published:** 2017-04-21

**Authors:** Ning Yang, Meng Li, Jörg Patscheider, Seul Ki Youn, Hyung Gyu Park

**Affiliations:** 1 Nanoscience for Energy Technology and Sustainability, Department of Mechanical and Process Engineering, Eidgennössische Technische Hochschule (ETH) Zürich, Tannenstrasse 3, Zürich CH-8092, Switzerland; 2 Laboratory for Nanoscale Material Science, Empa (Swiss Federal Laboratories for Materials Science and Technology), Überlandstrasse 129, Dübendorf CH-8600, Switzerland

## Abstract

A precise control of the dimension of carbon nanotubes (CNTs) in their vertical array could enable many promising applications in various fields. Here, we demonstrate the growth of vertically aligned, single-walled CNTs (VA-SWCNTs) with diameters in the sub-1.5-nm range (0.98 ± 0.24 nm), by engineering a catalyst support layer of alumina via thermal annealing followed by ion beam treatment. We find out that the ion beam bombardment on the alumina allows the growth of ultra-narrow nanotubes, whereas the thermal annealing promotes the vertical alignment at the expense of enlarged diameters; in an optimal combination, these two effects can cooperate to produce the ultra-narrow VA-SWCNTs. According to micro- and spectroscopic characterizations, ion beam bombardment amorphizes the alumina surface to increase the porosity, defects, and oxygen-laden functional groups on it to inhibit Ostwald ripening of catalytic Fe nanoparticles effectively, while thermal annealing can densify bulk alumina to prevent subsurface diffusion of the catalyst particles. Our findings contribute to the current efforts of precise diameter control of VA-SWCNTs, essential for applications such as membranes and energy storage devices.

Diameter control of vertically aligned carbon nanotubes (VA-CNTs) in the sub-2-nm regime is an exciting challenge. The architecture of vertical arrays of conductive hollow nanotubes has allowed envisioning various applications such as CNT membrane, supercapacitor, passive heat exchanger and sensors^1^,^2^,^3^,^4^. Structure-induced performance of these devices lays strong emphasis on the precise control of the geometry of VA-CNTs including the nanotube diameter. Particularly for single-walled carbon nanotubes (SWCNTs) in the applications of electronics and nanofluidics, the diameter is strongly concerned with the band gap and the transport enhancement, respectively.

Despite the great advances in the past years, shrinking the diameters of VA-CNTs below 2 nm in a reliable economic way is still challenging for two primary reasons. First, tiny metal catalyst nanoparticles get thermally unstable and vulnerable to coarsening at temperatures of chemical vapor deposition (CVD). The catalyst nanoparticles are usually formed by dewetting a metal thin film, e.g., Fe, on a metal oxide support layer, e.g., alumina^5^,^6^,^7^. At elevated temperatures, Fe nanoparticles can dewet into islands on the support in order to minimize the surface free energy at the interface they make with the alumina of lower surface energy. The thermal energy at CVD process could also result in coarsening of nanoparticles, especially the smaller ones^8^. The morphological change of the nanoparticles has a direct influence on the diameters of as-grown CNTs by increasing the average value and broadening the distribution. Second, the tinier the catalyst nanoparticles, the more susceptible they are to carbon poisoning (i.e., choking, carbide formation, etc.) and subsurface diffusion. Since individual CNTs are collectively grown in the VA-CNT synthesis, a local drop of the CNT areal density below a critical threshold could lead to crumbling of the vertical alignment and self-termination of the VA-CNT growth^9^,^10^,^11^. Thus, the carbon poisoning and the subsurface diffusion are the two main mechanisms responsible for the declination of the nanotube areal density and the cease of growth. As a result, the vulnerability of nanoparticles to such factors as coarsening, amorphous carbon encapsulation, and subsurface diffusion, has been curtailing the effective VA-CNT growth in the sub-2-nm regime.

To obtain ultra-narrow VA-CNTs, researchers have investigated a wide ranges of parameters, primarily focusing on three approaches: pre-growth conditioning, CVD parameters, and catalyst engineering^8^. The catalyst conditioning involves the manipulation of the nanoparticle dewetting and reduction conditions such as H_2_ partial pressure, temperature ramp rate, and exposure time^10^,^12^,^13^,^14^,^15^. The CVD parameters comprise growth conditions such as growth temperature, carbon exposure duration, precursor gas composition and dwell time, moisture additives, and so forth^16^,^17^,^18^,^19^,^20^,^21^,^22^. For instance, by balancing the CVD temperature, limiting the C_2_H_2_ partial pressure, and rendering catalyst nanoparticles well isolated and sufficiently dense, Youn *et al*. have reported sub-2-nm-wide CNTs on an iron-on-alumina substrate^23^.

Other than the CVD conditions, researchers have long been interested in the catalyst engineering, given the fact that the CNT CVD is a heterogeneous catalysis process. One way of engineering the catalyst is to optimize the preparation method and tailor the composition such as mono- and bimetallic systems^24^,^25^,^26^. It is not only important to select the right catalyst material but also to prepare the catalyst properly in order to provide the desired nanoparticle morphology and stability. Several research groups have proposed to deposit nanoparticles as catalyst that are small and dense enough to minimize the CNT diameter^27^,^28^,^29^. Another way of tailoring the nanoparticle size is related to catalyst support engineering. Of the support materials available, alumina and silica are most widely used in the CNT growth. It is observed that CNTs grown on an alumina-supported catalyst have shown the fastest CNT nucleation rate and small diameters with narrow distributions^7^,^30^, while CNTs formed on a silica-supported catalyst require longer time for nucleation, large diameters, and low areal number densities^5^,^6^.

A number of recent studies have shed light on the significant role of the alumina support engineering in tailoring the CVD synthesis of VA-CNTs for their growth rate and SWCNT specificity. Noda *et al*. have proposed that alumina can help facilitate the CNT growth rate by adsorbing carbon precursors on its oxide surface to supply them to catalyst nanoparticles^31^. In addition, it has been suggested that alumina prepared in different techniques can lead to diverse lifetime, activity, and evolution of catalyst during the CNT growth^32^. Moreover, crystallinity of the alumina has also been reported as an important parameter. Amorphous alumina having high porosity has been preferred and able to result in good yield and fast growth, while single crystalline sapphire has been recognized as an “inactive” support. In light of modifying the alumina property, energetic ions bombarding onto a single-crystalline sapphire layer has been shown to turn the exposed surface into a porous layer, usable as an active catalyst support for the VA-CNT growth^33^,^34^. Bearing a potential practicability issue for large scale production, the effect of this method on the resultant nanotube diameters remains to be elucidated. An O_2_ plasma treatment of alumina has been reported to help inhibit the subsurface diffusion of Fe into alumina, resulting in the large areal density and prolonged activities of the Fe nanoparticles^32^. Modification of the acidity of alumina has also been observed to have an influence on the CNT growth lifetime, efficiency, and the diameter of multi-walled VA-CNTs^35^. Moreover, it has been reported that by adding a very thin layer of alumina over an Fe oxide, an alumina-Fe-alumina sandwich structure could yield small-diameter CNTs with high purity and millimeter-scale lengths^36^,^37^,^38^. The anticipated role of the top surface of alumina is to immobilize nanoparticles during the growth, although a thorough investigation of the underlying mechanism is absent. Despite the notable improvement in understanding the role of alumina in the CNT CVD growth, only a few reports have focused on the relation between the alumina support properties and the VA-CNT diameters, particularly in the SWCNT diameter regime, and therefore it is still challenging to achieve a SWCNT forest in a sub-2-nm diameter range.

Here, we demonstrate the growth of VA-SWCNTs with diameters in a sub-1.5-nm regime by engineering the properties of the catalyst support, i.e., alumina. We first hypothesize that high porosity at the surface and high density (low porosity) in the bulk alumina could inhibit the surface coarsening and the subsurface diffusion of the catalyst nanoparticles. Then, we design a sequential procedure for the alumina treatment that conjoins thermal annealing and ion beaming, with which to grow VA-SWCNTs of ultra-narrow diameters. To understand how this sequential treatment helps safeguard this growth, we analyze the effectiveness of each process and the sequence in a decoupled way. Based on the findings, we explain the mechanism of engineering the catalyst support for growing a forest of sub-1.5-nm-wide SWCNTs.

## Results and Discussions

Our overarching goal is to understand and optimize the properties of the alumina support to grow the ultra-narrow VA-CNTs. Thermal dewetting of metal thin films into nanoparticles is selected for the catalyst preparation method throughout this investigation. If the dewetted catalyst nanoparticles shall disperse themselves finely, homogeneously and densely on the alumina support, and if they shall keep the dispersion without agglomeration or subsurface diffusion during CVD, the alumina underlayer has to be porous at the surface but needs to remain densified in subsurface zones. Otherwise severe subsurface diffusion could possibly deteriorate the vertical alignment^38^, particularly for the growth of narrow nanotubes. Although desired, the task of preventing both the agglomeration and the subsurface diffusion of the catalyst nanoparticles is challenging because of the inverse correlation between the porosity and density of the alumina support.

**Figure 1:**
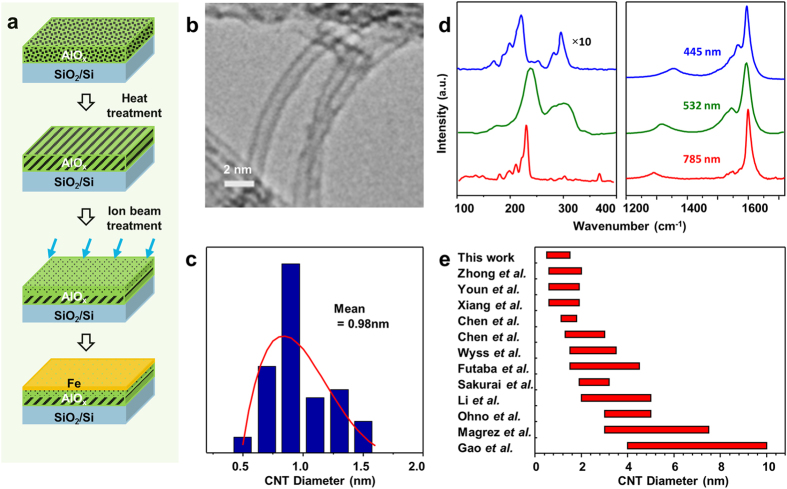
Sequential treatments of alumina and the as-grown sub-1.5-nm VA-CNTs. (**a**) E-beam deposited alumina is brought to and kept at high temperature of 800 °C for 30 min and then bombarded by Ar^+^ beam of 600 eV for 1 min before 1-Å-thick (nominal thickness) Fe catalyst is deposited. (**b,c**) The transmission electron microscope image (scale bar: 2 nm) and the histogram of the as-grown nanotubes show an average mean diameter of 0.98 nm and upper bound of 1.5 nm. The nanotubes are single walled. (**d**) Raman spectra of the as-grown VA-CNTs for the excitation wavelengths of 785 nm (red), 532 nm (green), and 445 nm (blue). (**e**) Diameter distribution of VA-CNTs obtained by the present and previous reports^10^,^17^,^18^,^25^,^26^,^28^,^32^,^35^,^36^,^37^,^51^,^52^.

To overcome this challenge, we have conceived a procedure that decouples the control of bulk and surface properties of the alumina support. This procedure utilizes thermal annealing and ion (Ar^+^) beam treatment to densify the bulk alumina and to change the surface properties, respectively (see Methods for details). A sequential procedure of annealing and ion beam treatment is designed prior to the Fe catalyst deposition (Fig. 1a). As the result, the Fe catalyst supported by the thus treated alumina layer produced VA-SWCNTs with diameters ranging between 0.5 nm and 1.5 nm (Fig. 1b,c). Raman spectra (Fig. 1d) has full width at half maximum of G band is 26 cm^−1^, and the G-to-D band intensity ratio is 12, which presents the typical features of high quality SWCNTs^39^,^40^. The spectra at three excitation laser wavelengths (445 nm, 532 nm, and 785 nm) show prominent radial breathing modes (RBM) between 170 cm^−1^ and 300 cm^−1^. The chirality map is deduced from RBMs (Supplementary Information), designating the existence of CNTs in the diameter range between 0.8 to 1.5 nm, in excellent agreement with the TEM characterization result of the nanotube diameter distribution. According to the chirality map, one third of the spotted CNTs are metallic, indicating random chiral distribution of the SWCNTs^41^. In contrast, the control sample of as-deposited alumina, to which no treatment had been applied, yielded no vertical alignment despite using the identical catalyst and the CVD condition. This result underpins a critical role of the alumina treatment in the growth of ultra-narrow VA-CNTs. In comparison to the previous reports, the SWCNT diameter distribution we obtained lies among the smallest (Fig. 1e).

In order to understand the effect of each treatment on the growth of the SWCNT forest, we compared growth performance (i.e., diameter distribution and length of as-grown nanotubes) on four types of alumina supports: (a) as-deposited (control); (b) annealed only; (c) ion-beam-treated only; and (d) annealed and ion-beam-treated sequentially. Except for the support preparation method, the other conditions of Fe catalyst deposition (2 Å by nominal crystal-monitor reading) and CVD (see Methods for details) are identical for various runs unless otherwise specified.

**Figure 2:**
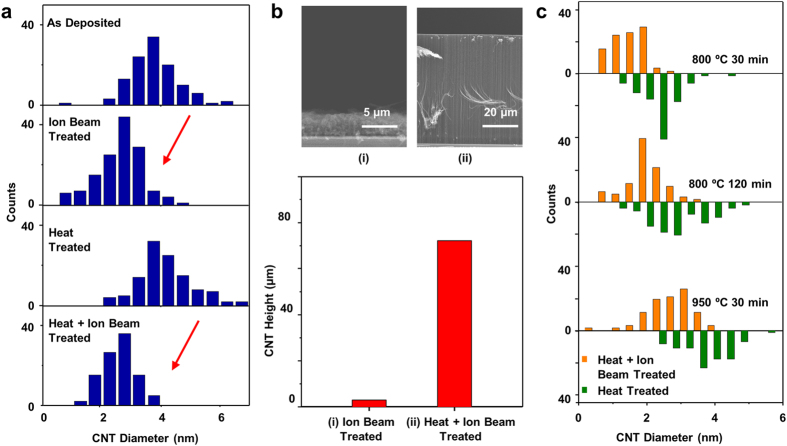
Impact of alumina treatments on CNT diameter and height. CNTs are grown by identical Fe catalyst of 2 Å under same CVD condition on different alumina supports. The diameter distribution of CNTs characterized by TEM images are shown in (**a**). Ion beam treatment applied on alumina substrate results in a reduction of CNT diameter, as shown by the red arrows in (**a**); heat treatment applied on alumina leads to slight increase of CNT diameter. The height of CNTs after 5 min of growth on two alumina samples, ion beam treated (4 μm) and heat plus ion beam treated alumina (77 μm), are characterized by SEM images and are compared (**b**). The impact of heat treatment condition on diameter of CNTs is illustrated in (**c**), by comparing the diameter distribution of CNTs grown on heat + ion beam treated (orange) and heat treated alumina (green). Three heat treatment conditions are applied to alumina: 800 °C 30 min, 800 °C 120 min, and 950 °C 30 min.

TEM analysis reveals that the ion beam bombardment on alumina leads to small nanotube diameters, whereas thermal annealing has an opposite effect. When the pristine alumina support was bombarded with Ar^+^ beam (see Methods for details) prior to the Fe deposition, the mean diameter of the resultant CNTs decreased from 3.7 nm to 2.6 nm; similarly, when the annealed alumina support was bombarded with Ar^+^ beam, the resultant CNT diameter dropped from 4.1 nm to 2.5 nm (Fig. 2a, red arrows). On the other hand, the thermal treatment resulted in slight enlargement of the CNT diameter. Besides, it turns out that the sequence of the alumina treatment is critical for the nanotube growth rate and the number density; a sequence of annealing prior to the Ar^+^ bombardment led to the growth of VA-CNTs, while the reverse sequence ended up growing short and poorly aligned CNT brushes underlying a random CNT network, likely caused by the growth termination at an early stage (Fig. 2b). These evidences bolster the effectiveness of our approach for the formation of densely distributed, tiny catalyst nanoparticles towards the ultra-narrow SWCNT forest growth.

However, excessive annealing of alumina for a prolonged duration or at excess temperature deteriorates the effectiveness by causing an undesired increase of the resultant CNT diameters. We compared the diameter of VA-CNTs grown on the Fe catalysts supported by three differently annealed alumina: (A) 800 °C for 30 min, (B) 800 °C for 120 min and (C) 950 °C for 30 min with or without additional ion beam bombardment (Fig. 2c, orange and green histograms, respectively). Excessive annealing conditions like (B) and (C) broaden and enlarge the CNT diameters, compared with the case (A) undergoing moderate annealing. A moderate thermal annealing is as critical as the ion bombardment for narrowing down the diameters of VA-SWCNTs. For all the annealing conditions, on the other hand, the effectiveness of the ion bombardment towards the CNT diameter shrinkage is reconfirmed (Fig. 2a).

**Figure 3:**
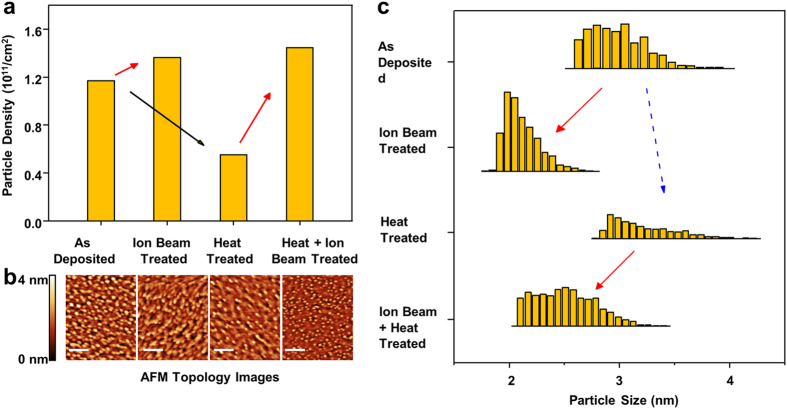
AFM characterization of catalyst nanoparticle density and size on various alumina supports. The sample with Fe nanoparticles was directly cooled down in Ar after dewetting in H_2_/Ar mixture without carbon exposure. (**a**) Density of as formed nanoparticles. The particle density is lower on heat treated alumina (black arrow) and higher on ion beam treated and heat + ion beam treated alumina (red arrows). (**b**) Topology images of catalyst substrates measured by AFM. The scale bar is 100 nm. (**c**) Size distribution of nanoparticles. The particle size decreases on alumina with ion beam treatment (red arrows) and increases on alumina with heat treatment (blue arrow).

To elucidate the formation and evolution of the catalytic nanoparticles on the alumina treated above, we characterized the surface morphologies (size and number density) of Fe nanoparticles formed atop four different types of the alumina supports using atomic force microscopy (AFM). AFM scanning measures the Fe particle heights of the Fe-on-alumina samples that had been annealed altogether in an H_2_/Ar atmosphere (see Methods for details) without exposure to C_2_H_2_. The resultant nanoparticle morphology represents the nanotube nucleation centers that have evolved on the surface from the initial thin film^42^,^43^,^44^. We observe that the Fe films deposited on the ion-beam-bombarded alumina convert to nanoparticles that are higher in density and smaller in diameter than those on the as-deposited or solely annealed alumina supports (Fig. 3a, indicated by red arrows). Alumina annealing alone, on the other hand, turns out to decrease the nanoparticle density (Fig. 3a, black arrow) and broaden the nanoparticle sizes (Fig. 3c, blue arrow). The AFM observations and the TEM analysis (Fig. 2) collectively support our claim that the alumina surface can influence the evolution of the catalyst particles responsible for the dimension of nanotubes nucleated from them. The AFM-resolved nanoparticle heights are slightly smaller than the corresponding CNT diameters in general, possibly attributable to imperfect hemispherical or elliptical geometry of the dewetted particles^45^,^46^.

**Figure 4:**
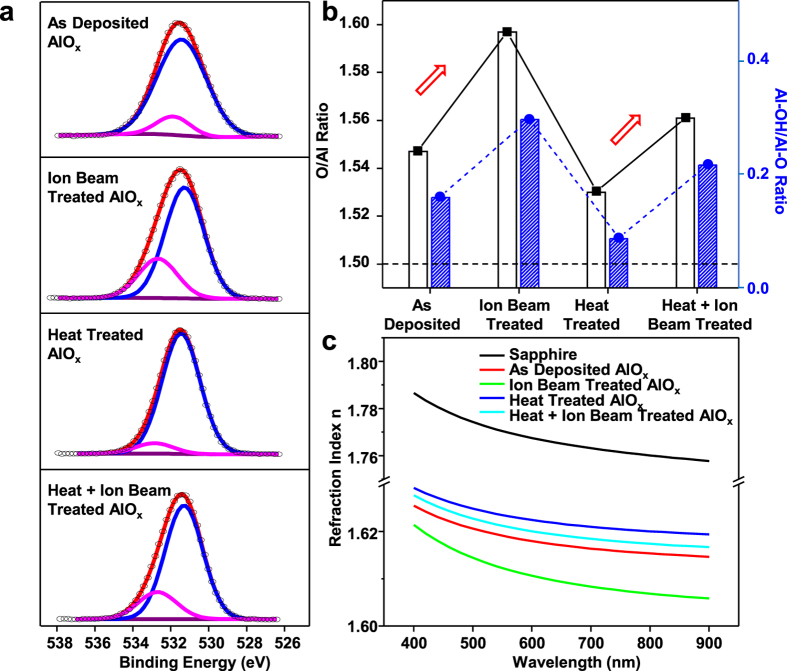
Comparison of four types of the alumina support for surface chemical composition and bulk density via X-ray photoelectron spectroscopy (XPS) and spectrometric ellipsometer. (**a**) To calculate the O/Al ratio, each O 1s spectra in XPS measurements are decomposed into contributions from Al-OH (magenta) and Al-O (blue). Such decomposition is shown for the O 1s spectra obtained at surface of as deposited, ion beam treated, heat treated, and heat + ion beam treated alumina. (**b**) The ratios of O to Al elemental composition (black, no pattern) and the ratios of Al-OH to Al-O (blue, slanted line pattern) in the alumina films based on the quantitative analysis of the O 1s and Al 2p core level spectra. (**c**) Index of refraction as a function of wavelength for various alumina supports. The curves were simulated to fit the Cauchy model. The crystalline sapphire (black) has the highest refractive index, indicating the lowest porosity. The as-deposited alumina (red) increases its refractive index upon thermal annealing but decreases after ion bombardment (green). The refractive index of sequentially treated alumina (light blue) lies in between those of the annealed and as-deposited alumina.

To understand the way how the thermal and ion beam treatments alter the properties of the alumina support, we carried out X-ray photoelectron spectroscopy (XPS) and spectroscopic ellipsometry. Since X rays have a penetration depth of only a few nanometers, XPS analysis is a suitable tool for probing the hydroxyl (-OH) content and the O-to-Al atomic ratio (O/Al ratio) on the surface and subsurface of the alumina. The O 1s core level peak of stoichiometric alumina is located at 531.4 eV (Fig. 4a, blue peaks). In addition to that, we observe shoulders by an additional peak at 532.5 ± 0.5 eV, an indicator for the existence of -OH bonds. Remarkably, this asymmetry introduced by -OH is the most conspicuous on the ion-beam-modified alumina and is hardly noticeable on the solely annealed one. In order to quantify the elemental composition in the topmost layer of the alumina support, we scaled the background subtracted, raw peak areas of the analyzed core levels to relative sensitivity factors (i.e., 0.66 for O 1s and 0.185 for Al 2p)^47^ and determined the ratio of incorporated oxygen to aluminum (O/Al ratio). All films are slightly oxygen-rich in comparison to stoichiometric alumina (Al_2_O_3_, O/Al ratio = 1.5). Particularly, the ion bombardment is found to enrich the surface oxygen content of alumina significantly (Fig. 4b, red arrows). As ion beam-treated alumina exhibits enhanced surface porosity, it also provides more adsorption sites for H_2_O, which is most likely the origin of the observed OH species. Annealing proves this hypothesis by bringing back the O/Al ratio towards the stoichiometric ratio. Finally, the sequentially engineered alumina (annealing followed by ion bombardment) exhibits a moderate change of the O/Al ratio, lying in between the annealed and ion bombarded alumina.

Scanning ellipsometry allows to characterize the optical properties of dielectric thin films; it can provide information about the changes in the bulk density (or porosity) of alumina because the refractive index is correlated positively (inversely) with the bulk density (porosity) of the thin film (Fig. 4c). Crystalline sapphire (black line) as a reference shows the highest density and refractive index. The refractive index of alumina decreases in the following order: sapphire (black) > annealed (blue) > annealed and ion beamed (light blue) > as-deposited (red) > ion beamed (green). This trend indicates that thermal annealing and ion bombardment can increase and decrease the apparent density of alumina, respectively. We speculate that the former treatment affects the bulk alumina density, whereas the latter induces defects near the surface^48^. Sequentially (annealing followed by ion bombardment) treated alumina is denser than as-deposited or ion beamed alumina yet less dense than annealed alumina. The relatively low bulk density of the sequentially treated alumina is attributed to the contribution of a porous surface layer. Excessive annealing further densifies alumina; the refractive index (bulk density) of alumina treated by various heat conditions decreases as follows: 950 °C 30 min > 800 °C 120 min > 800 °C 30 min> as-deposited (Supplementary Information).

To sum up, the ion beam treatment of alumina provides a structure with near-surface porosity leading to enriched -OH group and low apparent density (high porosity), while thermal annealing densifies alumina, with possibly removing physisorbed water (-OH) and reorganizing the atoms. The moderately enhanced value of the O/Al ratio in the sequentially engineered alumina is explainable by the cumulative effect of annealing and ion beaming. Upon annealing, for example, alumina is likely to be hardened and densified^49^.

**Figure 5:**
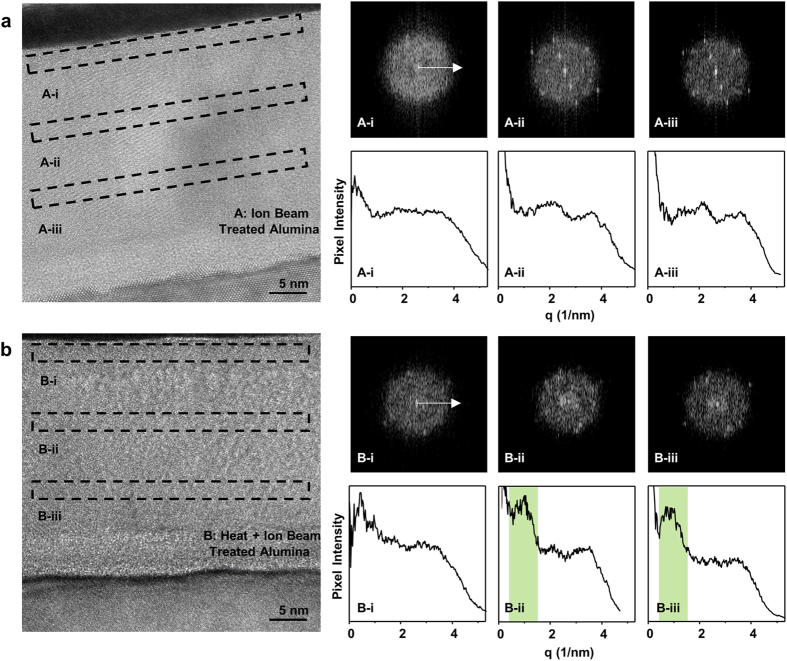
Cross section analysis of the alumina support layers via high-resolution transmission electron microscopy (HR-TEM) and fast Fourier transformations (FFT). The cross-section images marked with three selected areas representing the top, middle and bottom parts (height × width = 2 nm × 33 nm) and their fast Fourier transformation (FFT) images of (**a**) the ion-beam-treated alumina (type A) and (**b**) the annealed-and-ion-beam-treated alumina (type B). Both alumina thin films are ~33 nm and deposited on a Si (100) wafer. The pixel intensity profile with respect to radius (q, spacing in reciprocal space) is plotted by adding up all the pixel intensity at the same radius from the corresponding FFT images.

For an in-depth analysis of the potential phase change caused by the thermal annealing and of the penetration depth of the ion beam bombardment, we carried out high-resolution TEM and fast Fourier transformation (FFT) across the film thickness of the ion beamed alumina with and without the annealing pretreatment at 800 °C for 30 min (Fig. 5). According to the cross-sectional TEM images, both alumina samples are polycrystalline having crystalline domains of a few nanometers. For a detailed analysis, we selected three strips, each as thick as ~2 nm, across the alumina thickness to investigate via FFT their variation in the crystallinity with the film depth. For the ion beamed alumina without preannealing (Fig. 5a), FFT of the top strip (A-i) shows a ring pattern with no distinctiveness implying close to an amorphous phase; while the inner strips (A-ii, A-iii) exhibit recognizable spots in the pattern that hint a certain degree of a crystalline phase. The corresponding intensity profiles summed up along a q axis also show difference between the surface layer (A-i) and the bulk layer (A-ii, A-iii) in that more distinctive patterns are seen in the bulk. This observation indicates that the energetic ion bombardment used in this investigation indeed amorphizes the surface of the alumina support down to a few nanometers with leaving the subsurface layer intact.

For the ion beamed alumina with preannealing (Fig. 5b), FFT shows a diffraction pattern for all strips indicating a polycrystalline structure all over the alumina film, with no distinctive amorphous phase near the ion bombarded surface. The annealing-induced crystallization^49^ is indeed confirmed by the peaks in the intensity profile (e.g., B-ii and B-iii as opposed to A-ii and A-iii). In the intensity profile, a pattern at low q number observable for bulk strips (B-ii, B-iii) is different from the one for the surface strip (B-i). This observation suggests that the annealed alumina has a crystalline phase throughout the entire thickness, although the crystalline phase very near the surface may differ from that of the bulk to some extent.

**Figure 6:**
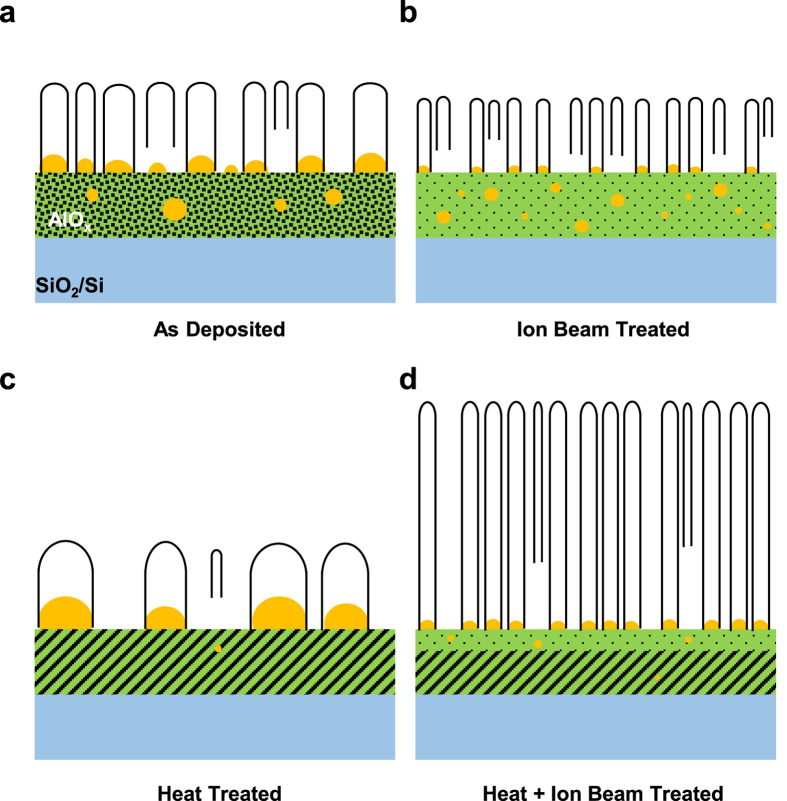
Schematics of the nucleation and evolution of catalyst particles on various alumina supports, including as-deposited (**a**), ion beam treated (**b**), heat treated (**c**), and heat + ion beam treated (**d**) alumina.

We schematically describe the Fe catalyst evolution and the related VA-CNT growth for the four variations of the alumina support, as follows. Prior to the CNT nucleation, the e-beam deposited Fe thin film dewets and transforms into catalytic nanoparticles at the catalyst-alumina interface upon exposure to the hot reducing atmosphere (see Methods for details). The density and the size of the resultant catalyst particles depend strongly on the surface energy (e.g., defects, porosity, etc.) of alumina. The ion bombarded alumina ends up bearing an amorphized surface layer, lowering the surface energy, and thus forming densely packed nanoparticles (Fig. 6b,d). In contrast, the annealed alumina becomes increasingly crystalline and densified, leading to high surface energy and thus prone to formation of enlarged nanoparticles at a relatively low number density (Fig. 6c).

After the dewetting stage, C_2_H_2_ is introduced into the chamber at CVD temperature of 800 °C for the CNT growth. The initial number density of CNTs is likely correlated with the initial number density of Fe nanoparticles, though the Fe nanoparticles located at the base of the growing CNTs can continue to evolve through Ostwald ripening and subsurface diffusion. The particle coarsening at the surface is the most severe for the annealed alumina and the least expressed for the ion bombarded alumina, possibly attributable to the high and low surface energies, respectively. The subsurface diffusion of the Fe atoms or clusters is predominant for the (solely) ion bombarded alumina, and can result in the deficiency of catalysts on the alumina surface, potentially deteriorating the CNT vertical alignment.

Finally, the ion bombarded alumina creating the low-energy surface layer enriched with -OH groups, atop which the Ostwald ripening can be desirably suppressed, can allow for the growth of ultra-narrow VA-SWCNTs (Fig. 6b); and if the ion bombardment is combined with proper preannealing of alumina, synergy could be achieved that enhances the areal number density and the catalytic activity of the catalyst nanoparticles but inhibits the otherwise vigorous subsurface diffusion, hence likely leading to sub-1.5-nm VA-SWCNTs (Fig. 6d).

## Conclusion

We investigated the optimal requirement for the alumina support towards the CVD growth of ultra-narrow VA-CNTs. By applying thermal annealing followed by ion beam treatment on the e-beam deposited alumina, we demonstrated the growth of sub-1.5-nm-wide VA-SWCNTs with a mean diameter of ~1 nm, which has not been obtained otherwise on the as-deposited alumina. Microscopic and spectroscopic characterizations reveal that the ion beaming on the alumina is effective in decreasing the catalyst particle sizes required for small SWCNT diameters, since it can amorphize and enrich the alumina surface with -OH groups to facilitate the formation of tiny nanoparticles. Through densifying the alumina, on the other hand, the annealing can enlarge the size of the catalyst particles yet inhibit the subsurface diffusion of Fe to sustain the CNT vertical alignment. These findings allow to conceive an alumina treatment condition optimal for the ultra-narrow VA-SWCNT growth, i.e., proper thermal preannealing followed by ion beam bombardment, eventually leading to one of the narrowest SWCNTs in a vertically aligned fashion to date on a Fe catalyst system, a breakthrough that can provide a guideline for the tailoring of CNT diameters. Our understanding of the underlying mechanism, if generalized, is potentially extendable to other transition metal catalyst systems for efficient VA-CNT growths for electronics and nanofluidics.

## Methods

### Preparation of alumina support layer

We deposited a 30-nm-thick alumina film on a Si (100) wafer by electron beam evaporation (Univex 500, Leybold) at a deposition rate of 0.2 Å s^−1^ at the chamber pressure in the range of 1–2 × 10^−6^ mbar. Two types of the postdeposition treatments were applied to the as-deposited alumina – thermal annealing and ion beaming – alone or in a combined way. The control group is the as-deposited alumina. The standard annealing temperature and duration are 800 °C and 30 min and have been varied for control experiments to be 800 °C for 120 min and 950 °C for 30 min, respectively. By bombarding the as-deposited alumina with Ar^+^ beam (Oxford Ionfab 300Plus) at 100 mA, 600 eV at an impingement angle of 20° for 1 min, we obtained the ion-beam-treated alumina. A sequentially treated alumina was obtained by annealing the as-deposited sample followed by the Ar^+^ beam bombardment. The sequence was reversed in order to test its effectiveness.

### Fe catalyst preparation and catalytic CVD

On the prepared alumina support, a thin layer of Fe is deposited via electron beam evaporation (2 Å by nominal crystal-monitor reading). During the deposition at 0.03 Å s^−1^, the chamber pressure was kept at 1–2 × 10^−6^ mbar. CVD of VA-CNTs was carried out in a cold-wall vertical reactor (Black Magic Pro^TM^, Aixtron AG) with a gas shower head. The dual heaters at the gas inlet and the sample stage and the cold-wall design allow fast heating and cooling.

Prior to growth, the chamber was cleaned by high flux H_2_ of 1000 sccm at 800 °C for 10 min. After the chamber was cooled down to 100 °C and below, catalyst substrates were loaded onto the sample stage. The sample stage was heated up at 200 °C per min by dual heaters to an annealing temperature and kept at the temperature for 40–60 sec. During the annealing, 200-sccm Ar and 800-sccm H_2_ were flown as a carrier gas and a reductant. The total gas pressure was kept at 8 mbar. Following the annealing, the chamber was quickly cooled down to and stabilized at 700 °C within 40 sec, before 5 sccm of C_2_H_2_ was added into the gas flow for the CNT growth. After the growth, the CVD reactor was cooled down in Ar, and the samples were taken out into the ambient below 400 °C.

### Characterizations

Pristine CNT forests were analyzed by scanning electron microscopy (Hitachi), high resolution transmission electron microscopy (Philips CM 12) at 100 keV, and Raman spectroscopy with three wavelengths of 785 nm (Renishaw RM 1000), 532 nm and 445 nm (WiTec CRM200). The atomic force microscope images were taken with an Asylum Research, MFP-3DTM in the tapping mode at a scan rate of 1 Hz and a cantilever oscillation frequency of ~279 kHz for a scan size of 1 × 1 μm^2^. A super sharp tip with a tip radius less than 5 nm (Nanosensors^TM^, SSS-NCHR) was used for an enhanced resolution. The images acquired were flattened to remove tilt in the image. In all measurements, nanoparticle size as opposed to width were measured because heights are unaffected by the tip radius. Characterization of the film chemical composition and stoichiometry was performed on alumina (AlO_x_) thin films on SiO_2_ by X-ray photoelectron spectroscopy (XPS) (PHI Quantum) in normal emission using monochromatic AlKα radiation (*hν* = 1486.7 eV) and a hemispherical electron analyzer. The refractive index of the alumina samples investigated by XPS was measured by a spectroscopic ellipsometer (SENTECH SE850). The measurements were carried out in a wavelength range between 400 nm to 900 nm. The angle of incidence was fixed at 70°. The change of refractive index indicates changes in the density of the films. A generic model was used with the following layer scheme: air/alumina/native oxide (silica)/silicon.


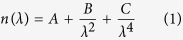



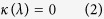


The refractive index of the overall alumina film was fitted using an equation of a Cauchy transparent layer^50^, which could be fitted to dispersion formulas to calculate the real (*n* in equation (1)) and imaginary (*κ* in equation (2)) parts of the complex refractive index. In eq. (1), *A* (dimensionless), *B* (nm^2^), and *C* (nm^4^) are material-specific constants influencing the curve fitting at long, medium, and short wavelengths (*λ*), respectively.

## Additional Information

**How to cite this article:** Yang, N. *et al*. A Forest of Sub-1.5-nm-wide Single-Walled Carbon Nanotubes over an Engineered Alumina Support. *Sci. Rep.* **7**, 46725; doi: 10.1038/srep46725 (2017).

**Publisher's note:** Springer Nature remains neutral with regard to jurisdictional claims in published maps and institutional affiliations.

## Supplementary Material

Supplementary Information
