# Supplementary material for: A Forest of Sub-1.5-nm-wide Single-Walled Carbon Nanotubes over an Engineered Alumina Support
Source: Sci Rep. 2017 Apr 21;7:46725. doi: 10.1038/srep46725 (PMC5399450; doi:10.1038/srep46725)
Supplement: Supplementary Information [file srep46725-s1.pdf]

Supplementary Information

A Forest of Sub-1.5-nm-wide Single-Walled Carbon Nanotubes over an Engineered Alumina Support

Ning Yang,[1] Meng Li, [1] Jörg Patscheider,[2] Seul Ki Youn, [1] and Hyung Gyu Park[1]**

[1] Nanoscience for Energy Technology and Sustainability, Department of Mechanical and Process Engineering, Eidgennössische Technische Hochschule (ETH) Zürich, Tannenstrasse 3, Zürich CH-8092, Switzerland

2 Laboratory for Nanoscale Material Science, Empa (Swiss Federal Laboratories for Materials Science and Technology), Überlandstrasse 129, Dübendorf CH-8600, Switzerland

* Correspondence should be addressed to: youns@ethz.ch (S.K.Y.), parkh@ethz.ch (H.G.P.)

Chirality distribution of sub-1.5 nm CNTs grown on engineered alumina

We analyze the chiral distribution of sub-1.5 nm CNTs grown on sequentially treated alumina (Fig. S1). Radial breathing mode (RBM) of CNT, which corresponds to the expansion-contraction of the nanotube, is measured by resonance Raman spectroscopy at multiple laser wavelengths (Fig. 1d). By knowing the frequency of RBM, the diameters of single wall CNTs are estimated based on the relationship $V_{RBM} = 234/d + 10$[1]. Each CNT is associated with a unique pair of indices (n, m) that fingerprints its properties.[2] Raw data of chiral maps is listed in Table 1. The estimated diameter ranges from 0.77-1.45 nm, which is in line with the distribution characterized by TEM (Fig. 1b, c).

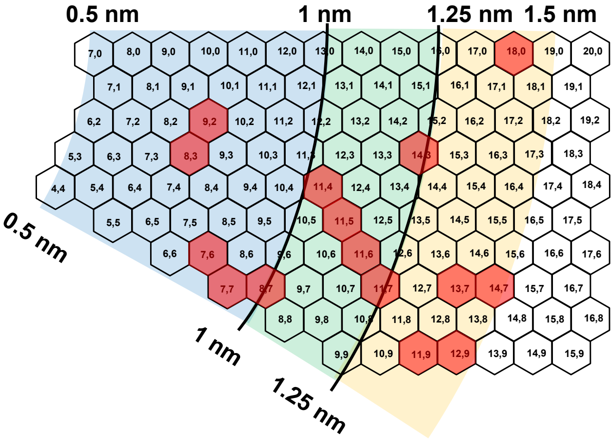

Supplementary Figure S1: Chiral map derived from radial breathing mode (RBM) wavenumber of grown sub-1.5-nm-wide single-walled CNTs grown on sequentially engineered alumina substrate with Fe catalyst of 0.1 nm.

n	m	d (nm)	V_{RMB} (cm^{-1})
8	3	0.77	313.42
9	2	0.79	304.45
7	6	0.88	275.17
7	7	0.95	256.48
8	7	1.02	239.87
11	4	1.05	232.12
11	5	1.11	220.78
11	6	1.17	210.11
11	7	1.23	200.14
14	3	1.23	200.14
11	9	1.36	182.25
13	7	1.38	180.00
18	0	1.41	176.02
12	9	1.43	173.76
14	7	1.45	171.36

Supplementary Table T1: Table of all assigned (n, m) for CNTs shown in S1. The diameter of CNT, d, is in nm, the frequency of RBM is in cm^{-1}.

Influence of heat treatment conditions on porosity (density) of alumina

In this section, we compare the porosity change at different heat treatment conditions using spectroscopic ellipsometry (Fig. S2). It is shown in Fig. 4c that heat treatment (at moderate condition of 800 °C for 30 min) densifies (reduces porosity) alumina. By fitting the SE data by the same method as Fig. 4c with Cauchy model, we summarize the deduced refractive index as a function of wavelength. We observe porosity increase in the following order: heat treated at 950 °C for 30 min< heat treated at 800 °C for 120 min< heat treated at 800 °C for 30 min (moderate condition) < Heat (800 °C for 30 min) + ion beam treated <as deposited (electron beam)< ion beam treated alumina. After heat treatment at 950 °C, alumina shows refractive index closer to sapphire. Generally, the catalyst nanoparticles experience an increase of Ostwald ripening rate increase with decreasing porosity.[3] This results implies that the distinct decrease in porosity after heat treatment could contribute to the serve diameter enlargement of CNTs grown on alumina treated at excessive conditions (800 °C for 120 min and 950 °C for 30 min).

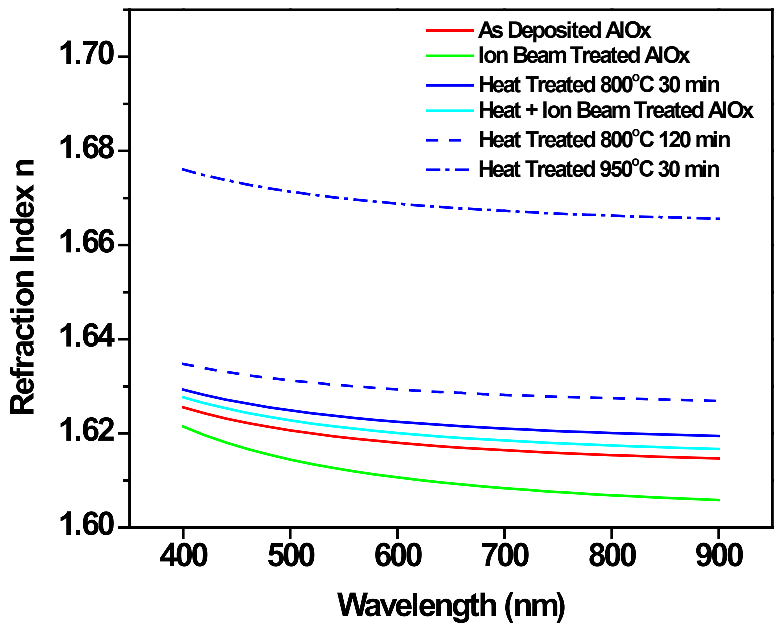

Supplementary Figure S2: Refractive index (n) of alumina sample with different heat treatment conditions as a function of wavelength. The curves were simulated to fit the Cauchy model. Temperature and time are two effective parameters of heat treatment. Alumina with heat treatment at 950 °C for 30 min (blue, dash-dotted line) has highest refractive index; followed by 800 °C for 120 min (blue, dash line) and 800 °C for 30 min (blue, solid line). Alumina with ion beam treatment (green line) has the lowest refractive index, lower than n on as deposited alumina (red line); whereas first heat and then ion beamed alumina (cyan) has n value, slightly higher than n of as deposited alumina.

Elemental mapping of the alumina thin film by electron dispersive spectroscopy (EDS)

To obtain elemental information of the engineered alumina, we performed high angle annular dark-field scanning TEM (HAADF-STEM) and EDS mapping during TEM measurements by FEI Talos F200X. We compared alumina with ion beam treatment (Fig S3. a) and with heat + ion beam treatment (Fig S3. b), and found very similar elemental distribution. Al and O are found spanning over 30 nm, corresponding to the alumina thin film. Below alumina dominant Si signal is observed, signalize the Si wafer. On top of alumina, a 5 nm layer of Au is detected. Au layer was deposited intentionally as a conductive layer to avoid charging effect of alumina during lamellar preparation by focused ion beam (FIB). On very top of the sample, we observe granular structure (from HAADF images) in both samples, which could be associated to the 100 nm of Pt layer coated in order to protect alumina from overheating and beam damage.

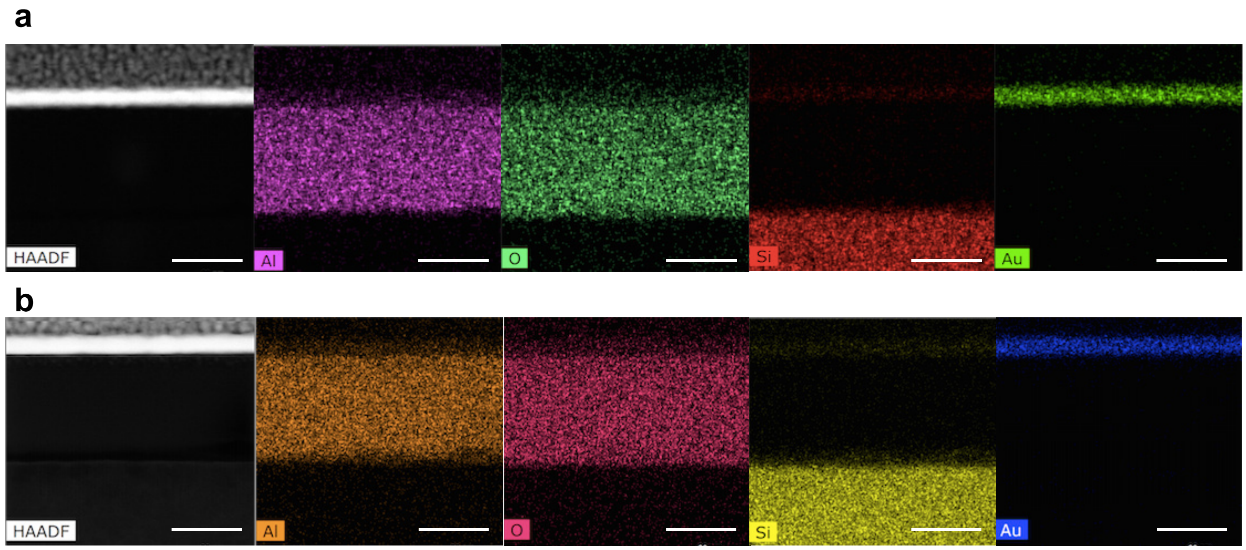

Supplementary Figure S3: Chemical mapping of alumina thin film cross-section by energy dispersive X-ray spectroscopy (EDS) analysis. Ion beam treated alumina (a) and first heat then ion beam treated alumina (b) show similar chemical composition across the layers. The characterization of element Al and O spanning over 30 nm evidences the presence alumina thin film. Below alumina is the Si substrate, whereas on top of alumina is the conductive coating layer of Au (5 nm).

Sequence of treatment on alumina as an influential factor for the structure of VA-CNTs. We reverse the treatment sequence of alumina, i.e. first ion beam treatment and then heat treatment, and compare the diameter and apparent growth rate of VA-CNTs respectively. (Fig. S4) CNTs grown alumina treated in regular order have average diameter around 1 nm and upper limit around 1.5 nm (a); while CNTs grown on alumina treated in reversed order have average diameter around 1.78 nm and upper limit around 4 nm (b). As discussed previously, ion beam treatment creates surface defects and increase porosity, whereas heat treatment heals defects and decrease porosity. For alumina with treatment in reversed order (b), surface defects and porosity created during ion beam treatment might be demolished by heat treatment, leaving the less defective and porous with high Ostwald ripening rate, contributing to large diameter and wider distribution in (b). The apparent growth rate, deduced from the height of CNTs grown after 5 min, is also lower in (b). The low surface porosity of alumina in (b) may results in large particles with low area number density, contributing to bad alignment of CNTs. In brief, to achieve effective growth of small diameter VA-CNTs, the sequence of treatments on alumina is critical and should not be altered. This results supports our model that heat treatment and ion beam treatment modify the bulk and surface properties of alumina respectively. By alternating the sequence, the impact of surface treatment will be weakened by the bulk treatment.

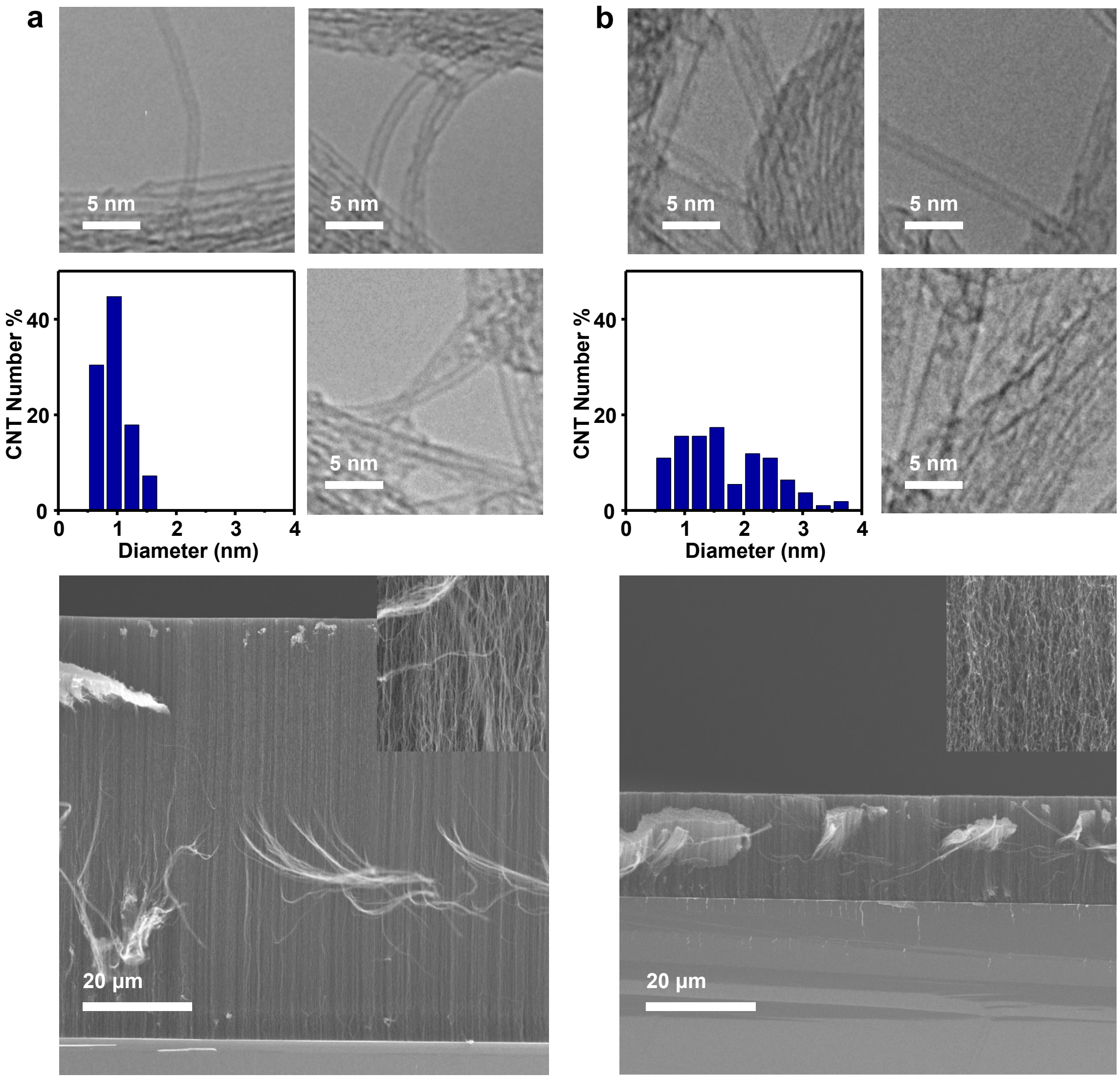

Supplementary Figure S4: Role of the treatment sequence on the structure of CNTs. Representative TEM and SEM images of CNTs grown on alumina support with first heat and then ion beam treatment (a) and on alumina treated in reversed order (b) that is first ion beam then heat. Compare CNTs grown on alumina (a) and (b), the prior one has small average diameter with narrower distribution, better vertical alignment as well as high apparent growth efficiency.

1. Dresselhaus, M. S.; Dresselhaus, G.; Saito, R.; Jorio, A. *Physics Reports* **2005,** 409, (2), 47-99.
2. Charlier, J.; Eklund, P.; Zhu, J.; Ferrari, A. *Carbon Nanotubes: Advanced Topics in the Synthesis, Structure, Properties and Applications*, Ed. by Jorio, A., Dresselhaus, G., Dresselhaus, M. S., **2008**, Springer.
3. Amama, P. B.; Pint, C. L.; Kim, S. M.; McJilton, L.; Eyink, K. G.; Stach, E. A.; Hauge, R. H.; Maruyama, B. *ACS Nano* **2010,** 4, (2), 895-904.
